# Factors influencing the decision to accept or decline aortic valve replacement for asymptomatic aortic stenosis: a nested longitudinal qualitative substudy of the EASY-AS randomised trial

**DOI:** 10.1136/bmjopen-2025-106485

**Published:** 2026-01-22

**Authors:** Peter Allmark, Bethany Taylor, Angela Mary Tod, Tony Ryan, Marc Dweck, Gerry P McCann, Anvesha Singh

**Affiliations:** 1 School of Allied Health Professions, Pharmacy, Nursing and Midwifery, University of Sheffield, Sheffield, UK; 2 British Heart Foundation Centre of Research Excellence and Edinburgh Heart Centre, Royal Infirmary of Edinburgh, University of Edinburgh, Edinburgh, UK; 3 Department of Cardiovascular Sciences, University of Leicester, Leicester National Institute for Health and Care Research Biomedical Research Centre, and Leicester British Heart Foundation Centre of Research Excellence, Leicester, UK

**Keywords:** QUALITATIVE RESEARCH, Valvular heart disease, Cardiac surgery, Cardiovascular Disease

## Abstract

**Abstract:**

**Objective:**

To examine how patients and family members decide whether to accept a highly invasive intervention (aortic valve replacement (AVR)) when their condition (aortic stenosis (AS)) is asymptomatic and its course uncertain.

**Design:**

Nested, longitudinal, qualitative substudy of an ongoing randomised controlled trial (RCT) (NCT04204915) testing early intervention (EI) versus watchful waiting (WW) in patients with asymptomatic severe AS.

**Setting:**

Six select UK sites of the RCT.

**Participants:**

Select participants of the RCT, their next-of-kin and some who declined RCT participation.

**Results:**

73 interviews were conducted, with 41 participants.

Few knew much about AS before diagnosis. Uncertainty and the need for reliable information regarding symptoms and progress was a significant problem.

While some expressed unease at a major intervention for an asymptomatic condition, there were no outright objections to the idea.

Those who declined participation in the RCT did so for personal reasons, for example, their home circumstances did not permit the required period of recovery or they felt too old to risk intervention.

Reasons for accepting early intervention included the belief that the condition was serious and likely to deteriorate, and so better to have the intervention before such deterioration, as well as avoiding long waiting lists. Trusting clinicians’ judgement played a part in some decisions. Patients also wanted choice in the type of intervention received.

The longitudinal interviews (n=32) showed satisfaction in the early intervention group despite some problems in the the early recovery phase, especially for those undergoing surgical AVR.

**Conclusions:**

Where evidence supports major intervention for an asymptomatic condition, patients are likely to accept the offer, although personal circumstances play an important role in decision-making. Where a condition is not well known to the public, such as AS, patients rely on clinicians and other resources to help decide. Liaison with patient groups in developing shared decision-making resources may help with complex decisions.

**Trial registration number:**

NCT04204915

**STRENGTHS AND LIMITATIONS OF THIS STUDY**

- The study is unique in using serial interviews with participants in a randomised controlled trial deciding whether to undergo a major cardiac intervention despite being asymptomatic.
- Its findings are relevant to other conditions requiring major intervention despite a low symptom load, such as early cancers or other cardiac valve conditions.
- The data included interviews with those who had declined participation in the main randomised controlled trial—this is an unusual and helpful addition.
- As a qualitative study, the findings are not generalisable; however, the sample was relatively large and included participants from multiple centres; the results are hence applicable to patients in healthcare systems similar to UK, but caution should be exercised when generalising to other regions.
- Limitations include sampling based on participant self-selection, leading to potential selection bias.

## Introduction

 Aortic stenosis (AS) is the most common valve disease requiring treatment in Europe and North America, and its prevalence is rising with an ageing population.^1^ The only available treatment is aortic valve replacement (AVR) either via open heart surgery (surgical aortic valve replacement (SAVR)), or the less invasive ‘keyhole’ procedure, transcatheter aortic valve implantation (TAVI).^2^ In the UK, the latter is presently offered mainly to those over 75 or at higher surgical risk.

Intervention is currently recommended once symptoms or cardiac dysfunction develop. However, AS is characterised by a long and variable asymptomatic course. As such, the timing of intervention in asymptomatic patients with severe AS remains highly controversial, with imaging studies demonstrating potentially irreversible myocardial changes, including scarring or ‘fibrosis’, even before the onset of symptoms.

Several small randomised controlled trials (RCTs) have recently demonstrated improved outcomes with early intervention,^3 4^ though these were in small, highly select populations. A more recent trial has reported no benefit.^5^ The ongoing Early Aortic Valve Intervention in Severe Asymptomatic Aortic Stenosis (EASY-AS) trial is the largest RCT to date that is currently recruiting asymptomatic patients with severe AS, and randomising them to early intervention or standard care.^6^ This trial tests the hypothesis that early intervention will result in better clinical outcomes and a cost reduction when compared with the conventional approach of expectant management or ‘watchful waiting’ until symptoms develop.

One feature of EASY-AS that makes it unusual, particularly from the viewpoint of participants, is that they are asymptomatic, but the trial intervention is highly invasive. Although the vanguard phase of the trial had confirmed that recruitment would be possible, we did not know what the attitudes of participants and their next of kin were to potentially having an AVR when asymptomatic, which may also have implications for any future implementation, if guidelines were to change.

This paper reports a qualitative patient experience substudy of the ongoing EASY-AS trial. The use of qualitative studies of this nature strengthens our understanding and insight into new interventions, as well as improving trial efficacy.^7^ The qualitative substudy here aimed to explore patients’ and family/caregivers’ perceptions of risk versus benefit, decision-making and the acceptability of AVR when asymptomatic, as well as any barriers to potential implementation of an interventional treatment strategy for asymptomatic patients.

## Method

### Study design

This was a nested, longitudinal qualitative substudy of the multicentre, multinational RCT, EASY-AS (NCT04204915) conducted at selected UK sites.^6^ The participants in EASY-AS are those with asymptomatic severe AS, with no symptoms such as exertional chest pain, significant dyspnoea, dizziness or syncope related to AS, as judged by their clinical teams. Participants in the main trial are randomly assigned in a 1:1 ratio to one of two arms: early intervention (‘EI’): AVR or TAVI, and standard care or ‘watchful waiting’ (‘WW’) until symptoms develop, following which they may be referred for intervention. The choice of SAVR or TAVI is based on clinical judgement and local services and is not influenced by participation in the trial. The WW group undergoes routine clinical follow-up, including regular echocardiography and symptom checks in outpatient clinics. As such, the trial is not of AVR versus no-AVR, but a strategy trial of early AVR versus AVR when and if symptoms develop.

### Participant selection and recruitment for qualitative substudy

**Figure 1:**
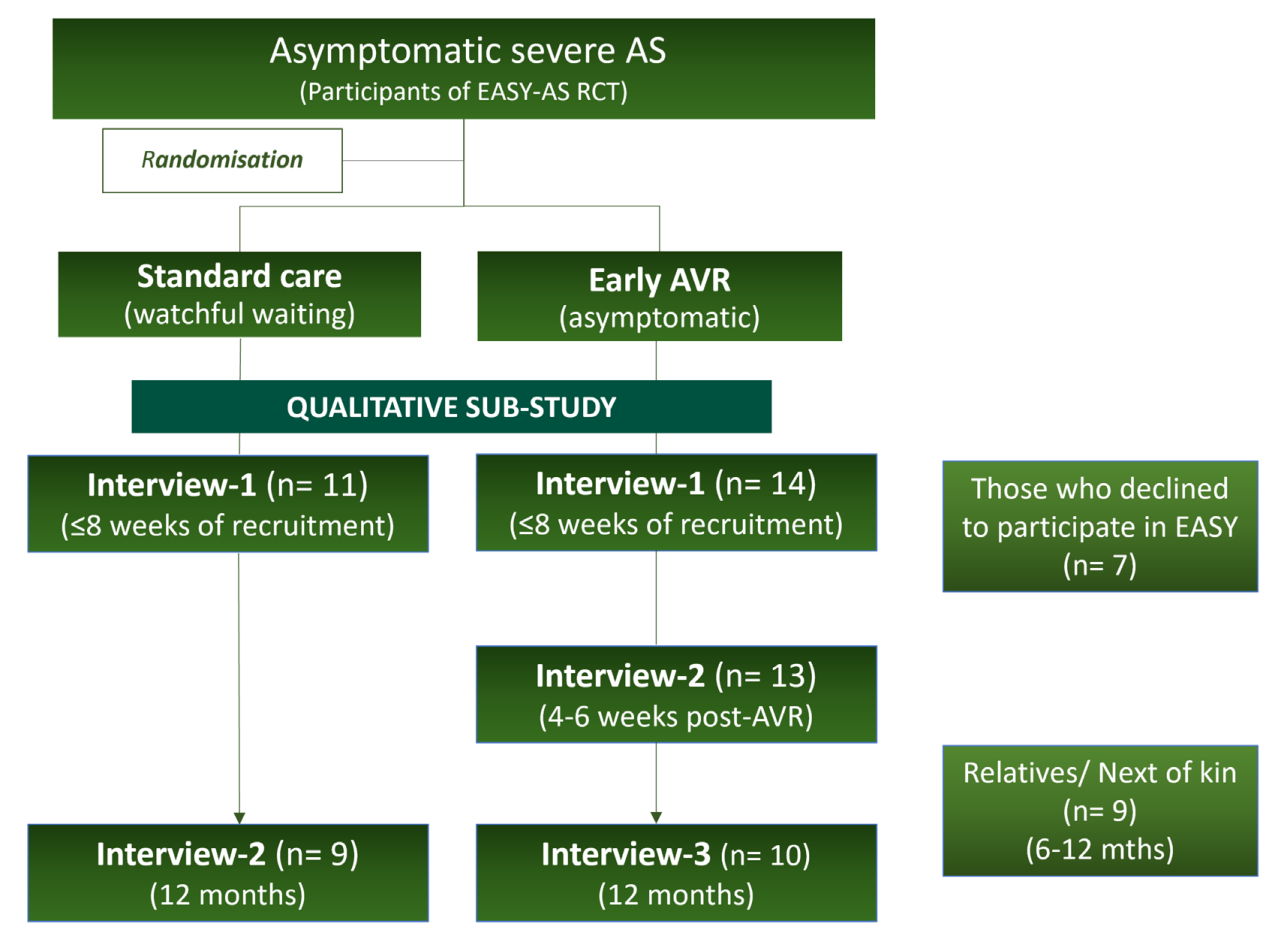
Recruitment flowchart for the qualitative substudy showing the number of interviews conducted in each subgroup. AS, aortic stenosis; EASY-AS, Early Aortic Valve Intervention in Severe Asymptomatic Aortic Stenosis; RCT, randomised controlled trials.

Those approached to take part in EASY-AS at six select sites in the UK (Leicester; Edinburgh; Wythenshawe, Manchester; Northumbria; North Tees and Hartlepool, and Middlesbrough) during the substudy recruitment period were given information about the qualitative substudy by the main trial team. This included some who declined participation (‘D’) in the main trial. Those interested contacted the substudy team directly by return of reply slip, who subsequently shared a patient information sheet with potential participants. The team then arranged written consent and interviews. Next of kin (‘NoK’) or significant others were also approached. Four groups of participants were invited to participate, shown in table 1. The substudy flowchart is shown in figure 1, which outlines the study design and interview schedule for the different groups. Longitudinal interviews were conducted with those who had been participants in the main study. The EI group were scheduled for three interviews: (1) within 8 weeks of recruitment to the main study; (2) 4–6 weeks post AVR; and (3) 12 months post AVR. The WW group were scheduled for two interviews: (1) within 8 weeks of recruitment; and (2) 12 months post recruitment. Only a single interview was conducted with those in the D and NoK groups. Next of kin were interviewed separately from the main participants.

**Table 1:** Groups of participants interviewed

Participant type	Acronym	Number
Patients randomised to Early Intervention in the EASY-AS trial	EI	14
Patients randomised to ‘Watchful Waiting’ in the EASY-AS trial	WW	11
Patients who Declined to participate in the main EASY AS trial	D	7
Family/caregivers or Next-Of-Kin of participants in the EASY-AS trial	NoK	9
Total		41

EASY-AS, Early Aortic Valve Intervention in Severe Asymptomatic Aortic Stenosis.

### Patient and public involvement

Patients with lived experience of valve and other cardiovascular disease were consulted during the design and funding application for this study, who shaped the design of this qualitative substudy. Following successful funding, a patient advisory group was formed, based on a best-practice framework,^8^ with help in recruitment by the Heart Valve Voice national patient charity. This consisted of five members of varying demographics from across the country, with lived experience of valve disease and/or valve intervention. This group provided input into all stages of the study, including recruitment strategy, development of the interview guides and analysis framework and comment on findings and their interpretation. They will continue to be involved in the dissemination of the results and planning future studies.

### Data collection and processing

One-to-one semi-structured interviews were conducted remotely, either by phone or web platform, by an experienced qualitative research associate (PA), independent of the main trial team, to encourage more openness and avoid bias. Interviews were conducted between 30 September 2022 and 9 April 2024. The interviews were based on topic guides developed with the patient advisory group (see online supplemental appendix 1 for an example). These guides had some iteration as the study went on. The interviews were audio recorded using an approved encrypted recording device and transcribed verbatim by an independent transcriber and all identifiable information removed. Once the transcripts were fully anonymised, the audio recordings were deleted.

### Data analysis

Framework analysis techniques were used for data analysis.^9^ These techniques enable the use of previous knowledge in the development and refinement of the thematic framework; in this, discussion with the patient advisory group was central. The anonymised transcripts were entered onto Quirkos V.2.5.3 for data organisation and coding. All were coded by one researcher (PA) with a 20% sample checked by two other researchers taking 10% each (AMT and TR). Coding began from an initial framework developed with the patient advisory group and the study team, then modified through iteration during analysis. There is some discussion in the literature on longitudinal qualitative research as to whether it is tied to a specific qualitative tradition. Some would, therefore, argue that framework analysis techniques are not compatible with it.^10^ However, this research project is probably best characterised as one which incorporates longitudinal (or follow-up) interviews to supplement the findings, rather than it being the central focus. We return to this point in the part of the results headed ‘Change over time’ below.

## Results

The results are presented here in four sections, with the key themes and subthemes summarised in table 2.

**Table 2:** Key themes and sub-themes identified

Theme	Subthemes/description
General data	Description of the cohort
Factors influencing decision	Receiving the diagnosis Knowledge of the condition
Making the decision	Acceptability of early AVR for asymptomatic AS Reasons for declining trial participation Reasons for accepting trial intervention and early intervention
Change over time	Allocation of treatment arm Experience of AVR Asymptomatic or not

AS, aortic stenosis; AVR, aortic valve replacement.

### General data

#### Interviews performed

A total of 73 interviews were performed with 41 participants, including follow-up interviews in the EI and WW groups (figure 1). Of the 25 interviewees that were also participants of the main randomised trial, 14/25 were assigned EI and 11/25 were assigned to WW. Of the EI group, 3 had TAVIs and the remainder had SAVR based on local clinical team decision. Not all planned follow-up interviews were performed. This was because of one patient withdrawing from the main trial and one withdrawing from the qualitative substudy. The remaining four had not yet had AVR or had delays in having AVR, resulting in insufficient time for the final interview to take place. All other interviews occurred as per the protocol. The average time taken for each interview was just over 22 min, ranging from 9 to 42 min.

#### Participant characteristics

The mean age of participants among the 25 interviewees who had consented to take part in the main trial (EI and WW groups) was 75 (range 54–83) years, and 14/25 (56%) were male. The mean age in group D was 74.3 (range 54–85) years, with 5/7 (71%) male subjects. The mean age of the NoK group was 63 (range 53–75) years, with 2/9 (29%) being male.

### Factors influencing the decision concerning early intervention

#### Timing of diagnosis

There is no routine screening for AS and the condition was asymptomatic in the trial participants. As such, diagnosis was usually by chance during investigation for another condition. In two cases, it was discovered when a relative who was a health professional had rested their head on the person’s chest and heard a heart murmur.

Occasionally, it was found when the patient was young. In these cases, they had been monitored for many years before they developed severe AS. The condition had not affected them greatly in the meantime. One interviewee said they had almost forgotten about it. Older patients who had been diagnosed with mild-to-moderate AS were also routinely monitored and none said they found the process onerous, although it could be at the back of their minds.

I: 20 years ago, when you were first diagnosed, did it play on your mind then?R: Yes, when I first found out that I had a dodgy valve, and eventually, they’re going to have to change it. But there’s more things in life to worry about. (P07, Int 1)

For others, the AS was already severe by the time it was diagnosed. For most patients, being told that their condition was now severe was an unpleasant surprise.

I moved from considering myself to be reasonably healthy four or five years ago, to being faced with major, major health issues, which has been difficult to take onboard fully, but I’ve got to. (D04)

As well as the patient’s own anxieties, the partner or next of kin may be concerned. There were also practical problems, such as with obtaining health insurance for holidays or with planning events.

#### Knowledge of the condition

Most patients had little knowledge of AS before diagnosis. The exceptions were those who had a background in healthcare or who had a family history of the condition. Once diagnosed, some investigated the condition either using an internet search or resources given to them by a clinician.

During the period following diagnosis, patients were told to self-monitor for symptoms. Not all, however, were clear what these might be, although most identified breathlessness as the main symptom to look out for. Others listed chest pain, dizziness and a possible collapse. Some patients were aware of the risk of sudden death.

Chest pain, shortness of breath, possible collapse. I think, probably, that was what we were advised to look out for… (C07)

Another issue for participants was the intrinsic uncertainty of the trajectory of severe AS; clinicians could not say for sure when the condition was likely to progress from asymptomatic to symptomatic. Those participants who were given a figure were told that it would be somewhere between 2 and 10 years, reflecting the variable course of asymptomatic AS.

### Making the decision

#### Acceptability of early AVR for asymptomatic AS

Analysis focused on the acceptability of AVR for people with asymptomatic AS. The interviewees were broadly categorised based on whether they found the idea acceptable (Y), not acceptable (N), were equivocal (E) or where no clear view was given (U) (table 3).

**Table 3:** Summary of how acceptable having a valve replacement for asymptomatic severe aortic stenosis was for participants

Early AVR acceptable? Yes (Y)/No (N)/Equivocal (E)/Unclear or No Comment (U)					
	Y	N	E	U	Total
Trial participants (EI and WW)	17	0	5	3	25
Next of kin	6	0	2	1	9
Trial decliners	1	1	4	1	7
Total (n (%))	24 (41.5)	1 (2.4)	11 (26.8)	5 (12.2)	41

AVR, aortic valve replacement; EI, early intervention; WW, watchful waiting.

None of the trial participants or next of kin said early AVR was unacceptable, although many were equivocal, for example, seeing that the intervention presented immediate risks while the risks of deferral were less certain and further off.

Being in hospital for a lengthy time or certainly longer than I’ve ever been in hospital before. And, you know, potential risks. I know you’re going to try and tell me now that the risks are very low but… D02

Not only did most trial participants view early AVR as acceptable, many (16/25) favoured the early intervention arm of the study. By contrast, two, both in the WW group, favoured the watchful waiting arm and would have considered withdrawing if they had been randomised to the early intervention arm. One of those who preferred the early intervention arm also said they considered withdrawing if they had not received it. In fact, only one of our interviewees withdrew from the study, and this was for reasons unrelated to views about the acceptability of early intervention.

#### Reasons for declining trial participation

We interviewed seven subjects who declined participation in EASY-AS (D01-D07). D02 and, to a lesser extent, D04 were supportive of the idea of early intervention but felt the risk was too high for them personally. For D02, it was other health issues that led him to decline the trial. His obesity, for example, made him believe the surgery would be of too high a risk. In contrast, D01 felt good for his age and did not want to put that at risk. Others in the decline group also had some concerns about the idea of prophylactic surgery, as they had no symptoms, so did not want to go through a high-risk procedure. Sometimes the decision to decline was related to age. D06 had a clearer objection in principle to the early intervention, that you should ‘not fix what is not broken’. This view was complicated, however, by an unhappy recent history of health interventions that had gone wrong and the subsequent unwillingness to trust in clinicians on the matter. A summary of the reasons given in the seven interviews is included in table 4.

**Table 4:** Main reasons for declining to participate in the EASY-AS randomised trial

Pt	Age	Sex	Summary of reasons from interview
D01	Early 80s	M	Feels okay for his age—does not want to mess with something not causing problems
D02	Early 70s	M	Obesity and ill health with worry about poor outcome; worry about effect on family
D03	Early 80s	M	Recent death of daughter/lack of understanding and focus
D04	Late 60s	M	Ran out of time
D05	Late 70s	M	No understanding of trial
D06	Early 50s	F	General attitude but also recent history of medical negligence proceedings
D07	Early 80s	F	Feels too unwell

EASY-AS, Early Aortic Valve Intervention in Severe Asymptomatic Aortic Stenosis; F, female; M, male; Pt, patient.

**Figure 2:**
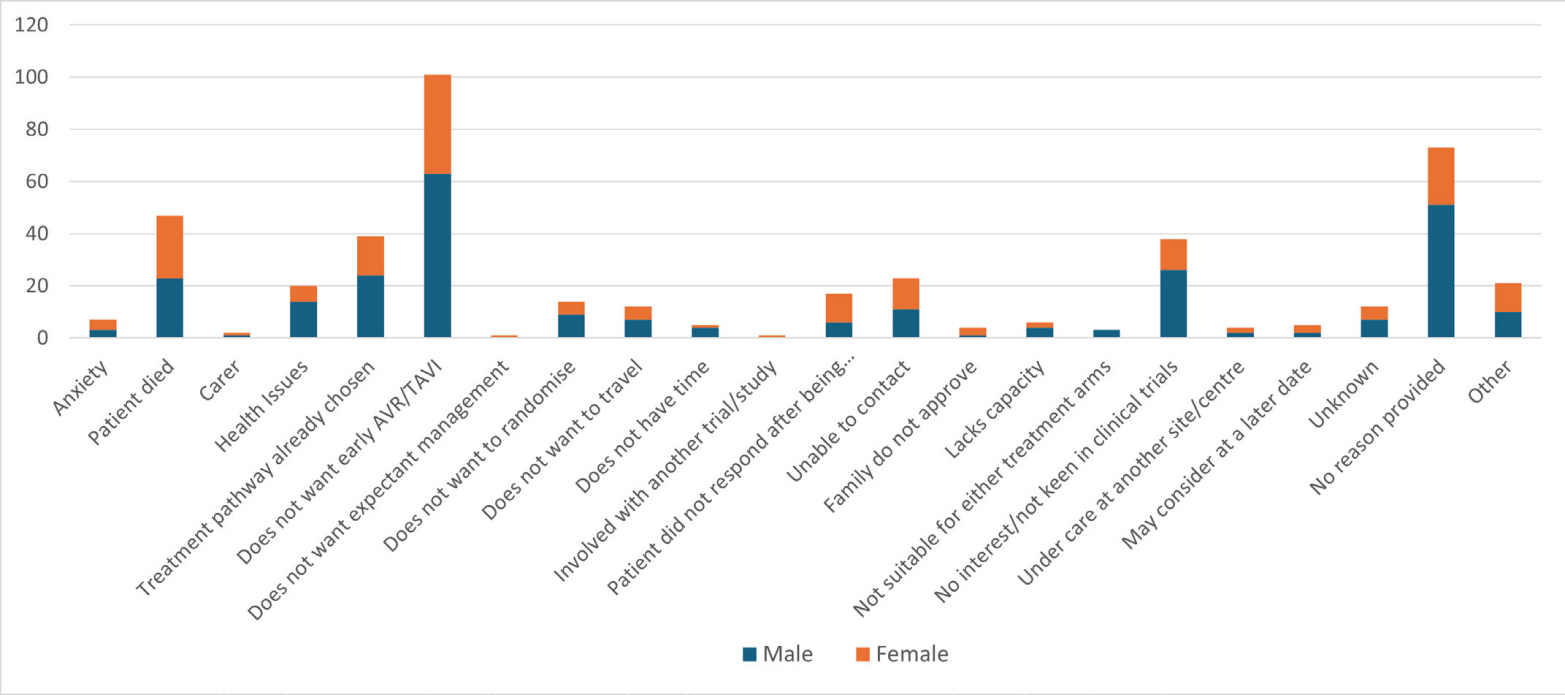
Reasons for declining participation in the main EASY-AS trial collected through UK-site screening logs. AVR, aortic valve replacement; EASY-AS, Early Aortic Valve Intervention in Severe Asymptomatic Aortic Stenosis, GP, general practitioner; TAVI, transcatheter aortic valve implantation.

The main EASY-AS trial also collects data on reasons for declining participation as part of the screening log (UK sites). At the time of writing, 898 men and 493 women were eligible, of whom 256 men (28%) and 166 (33.7%) women had declined to take part. The main reasons for declining participation in the main RCT are shown in figure 2, the most common reason being they did not want early SAVR/TAVI (n=101).

#### Reasons for accepting trial participation and early intervention

Some reasons for taking part in EASY-AS were trial specific. One of these was trial-focused altruism, the desire to help medical research. Another was the belief that being in a research study would bring benefits such as improved monitoring. Neither reason pertains to our focus in the substudy, which was the acceptability of early AVR to asymptomatic patients with severe AS. For that reason, we can set such trial-specific reasons aside.

#### Discussion with family

In most cases, the trial participants consulted with one or more next of kin or significant others. There were no examples of major disagreement within families although some unease was noted.

My daughter’s a bit bothered about it, she says, if you’re having heart surgery, mum, you could die on the table. I says, well, if I do, I do. (P11 Int 1)

Sometimes, however, the argument in favour of participation was given by a family member (often a health worker in addition); this could contribute to the participant’s decision to take part. A family history of heart valve (or other heart) problems sometimes contributed to the decision.

#### The serious nature of the condition

Many were persuaded that their condition, while asymptomatic, was serious and could become symptomatic and dangerous soon. Indeed, some said it could result in sudden and dangerous deterioration before it became obviously symptomatic, for example, causing black-outs while driving. In other cases, interviewees were convinced of the seriousness of the condition by their clinician, for example, by being shown their heart scan. In other cases, a family history of valve problems was enough to convince a patient of the seriousness of the condition.

#### Get it fixed while fit

Early intervention has the benefit of a relatively healthy patient rather than someone who has developed symptoms of illness; the former might be expected to recover better.

Yeah. My logic, and I think it was the same from my wife, was that I’m far better recovering from that surgery when I am feeling well, compared to feeling ill. (P13 Int 3)

There may also be a more general personality difference between those preferring to get something fixed before it causes problems and those holding the view that you should not mend what is not broken.

#### Am I really asymptomatic?

Some interviewees were not convinced that they were truly asymptomatic and hence hoped for improvement through intervention. The average age of the interviewees (excluding the NoK group) was around 75. Their condition was often picked up during the investigation for something else. As such, most participants had other health conditions, some of which had symptoms like those of AS, such as breathlessness, dizziness and feeling tired. Indeed, the ageing process itself may be associated with these symptoms.

I do get more tired than I used to, but I am getting older as well. I will sleep in the afternoon until [I] take myself to bed, but that is the only symptom really. (P20, Int 1)

Older patients may not undertake the level of activity required to bring on symptoms; one clinician is reported as telling a participant that they would probably develop symptoms if put on a treadmill. This created some uncertainty around assessing the true symptom status of patients.

#### Avoiding NHS queues

Some thought it would be better to have the intervention early rather than wait until symptoms presented and then join a National Health Service (NHS) waiting list. In some senses, this was a trial-specific reason as consent to the trial presented an opportunity to avoid future queues. However, it is possible that such pragmatic NHS-focused reasoning would still play a part in decisions about early intervention outside trials.

#### Choice of valve type

Some participants discussed the option of the type of valve, which is broadly either bioprosthetic or artificial/mechanical. The latter are usually offered to younger patients as they have a longer lifespan. However, recipients need to take the anticoagulant warfarin long-term. For this reason, some refused the option. For example, one participant had caring responsibilities for two family members and felt she could not manage the Warfarin timetable with that.

I’ve got Husband now [and I] look after my brother, as well…, if I was on Warfarin and I had that to fit into my timetable. (P19 Int3)

#### Choice of intervention route

TAVI did not seem to have been discussed with all patients although some read about it and raised it with their clinicians. In general, however, it was offered to older patients who met the criteria and, presumably, where it was available. Those offered it viewed it as positive. In one case only, a patient pushed for having a TAVI despite not meeting the criteria and it not being offered initially. Eventually, the patient received the TAVI within the trial, and it seemed he would not have gone ahead with open heart surgery.

They kept saying that the TAVI only lasted 15 years, so you said, well I’m 74, so yes. (P01 Int 2)

#### Trust in clinicians

Only one interviewee (D06) mistrusted clinicians, for reasons set out above. Many seemed trusting and some gave this as a reason for consenting to the trial. This was particularly the case where there was a pre-existing relationship from an earlier diagnosis and monitoring of AS.

There’s a trust and respect I have for what they’re doing and what they understand of what they’re seeing on the tests that they’ve made. So it’s not an out of the blue thing. It’s come out of a relationship with the hospital as well. (P12 Int 1)

### Change over time

This was not a formal longitudinal study, where change is a central focus of analysis.^10^ In this study, the follow-up interviews were held with EI and WW participants to supplement and triangulate findings, rather than explicitly monitor change. As well as contributing to the main findings of this study, they enabled us to track whether participants’ views changed or were maintained over time. In terms of our focus on the acceptability of AVR in asymptomatic AS, the following were of note.

#### Allocation of treatment arm

In the WW group, most who had expressed a treatment arm preference for either EI or WW at the first interview maintained it at the second. One WW participant, in their second interview, expressed some doubts about whether they would go ahead with the intervention. In the EI group, one participant said that in retrospect they were glad to have had the early intervention, having been in equipoise at the first interview; at least one other noted that their initial doubts were allayed by experience.

#### Experience of AVR

Those who had received a TAVI commented on the rapid recovery; those who had SAVR often commented on complications, pain and a slow recovery. The postoperative complication most commented on was pain from the scar, which usually resolved by the third interview. Other complications were arrhythmias and pericardial effusion. No one in the EI group, however, stated an unequivocal regret that they had received the early intervention. Further, when they did recover, physically it would only be to feeling as they had done before. Psychologically, however, some reported reassurance in knowing they were no longer at risk of sudden deterioration.

Sometimes I think to myself, would you have gone through this operation if you knew what was coming? And sometimes I’ll say to myself, no, I wouldn’t have. And then I say, yes, I would… I probably would have. (P6, Int 3)

#### Asymptomatic or not

It was noted above that some participants did not believe themselves to be asymptomatic at the time of intervention. This showed itself further in patients who noted an improvement in their symptoms.

I feel like they tapped me on the head with a wand and all my problems, such as short of breath, phlegm on the chest, walking, has all disappeared. (P18, Int2)

## Discussion

The substudy was nested in the ongoing EASY-AS RCT. Its focus was the acceptability of AVR, a major intervention, to patients who were asymptomatic and with a condition (severe AS) with a variable and uncertain course. This focus is of interest primarily to clinicians who would be implementing early AVR if it is shown to be effective, and the findings may also be relevant to other asymptomatic conditions requiring invasive treatment. To our knowledge, no previous study has addressed this specific issue.

### Previous studies

There are relevant studies in other areas where watchful waiting (or active surveillance) has been compared with an intervention, including surgical ones. Often these relate to cancer, most famously the ProtecT trial.^11^ This compared three treatments for prostate cancer: active monitoring, surgery or radiotherapy. All three showed similar outcomes although the invasive treatments reduced cancer progression over time. There have also been trials in areas such as renal^12^ and oesophageal cancer.^13^ The latter was a nested qualitative study; it noted a difference in mindset between patients who preferred active monitoring (‘enjoy life now’) and those who preferred immediate treatment (‘don’t give up, act now’). These are similar to the findings in our study where individuals’ circumstances and perception of quality of life played a role in their preferences. There have also been studies of surveillance versus surgery relating to gallstones^14^ and gut surgery^15^ in the frail elderly. However, neither study included a qualitative component.

It is noted that at least three considerations reduce the applicability of these studies. First, unlike the EASY-AS trial, the surveillance has usually been introduced as the new treatment where the active intervention was the standard. Second, where the condition is cancer, most people will have some pre-existing knowledge and concern that makes them predisposed to accept intervention, even major surgery. And, third, in many such studies, the patients are not asymptomatic.

### Consent to the EASY-AS RCT does not in itself imply the acceptability of early intervention

Successful recruitment to the EASY-AS RCT might suggest that AVR while asymptomatic was acceptable to those who consent to the trial. However, consent to RCTs is likely to be affected by specific factors. In the interviews, reasons included the desire to help medical research, avoid NHS queues and the belief they would receive better management within an RCT. These reasons would not transfer beyond the context of an RCT; in clinical practice, AVR for asymptomatic patients might be less acceptable.

### Declining EASY-AS does not imply belief that early intervention is unacceptable

At the time of this report, data from the main study showed that around a quarter of those who declined the RCT within the UK did so for reasons categorised as ‘does not want early AVR or TAVI’. The interview data indicates that in most cases this was not an objection to the intervention in principle but, rather, that it was not the right time for them. Participants in this substudy who declined the trial did so for reasons rooted in their personal or social circumstances.

Personal circumstances included seeing themselves as too old and unwell or, conversely, too old and well enough, and having significant comorbidities. Social circumstances included family bereavement or significant events, and responsibilities, such as caring for another. Research indicates that comorbidities can lead patients to dismiss early lung cancer symptoms, a tendency exacerbated by home responsibilities. This is found also in research concerning access to healthcare. For example, the presence of comorbidities alongside home responsibilities can exacerbate patients ‘explaining away’ emerging symptoms of lung cancer.^16 17^

### Consent to EASY-AS does suggest a belief in the acceptability of early intervention

By contrast, the reasons given by those who consented to the trial included many that were generic, that is, applicable across the broad group of asymptomatic patients with AS. Perhaps the key reason was that participants were persuaded of the potential serious nature of the condition despite being asymptomatic. Another reason commonly stated was that it would be better to undergo treatment early, while still relatively fit and before symptoms present. There was also an awareness that waiting for symptoms to present might then be followed by further delay on the waiting list for surgery. This reasoning may have been more powerful at the time of the interviews as it coincided with strikes and the aftermath of the COVID-19 pandemic.

### Choice of valve and intervention type mattered for some

The ability to be involved in decisions about treatment was important for some patients. Those young enough to be offered the mechanical valve were sometimes reluctant, mainly because of the need for ongoing warfarin treatment. The advantages of TAVI include lower peri-procedural risk and quicker recovery time than open heart surgery. The disadvantages include the need for re-do procedures, which can be more challenging. Its use, however, is widening, and many patients prefer this route. In the UK, however, there are resource and geographical variability challenges. In one case, a patient was insistent on a TAVI rather than SAVR even though he did not quite fit the local criteria. Other patients discussed the two treatments but were persuaded to take the treatment they were being offered. This links to the importance of trust in clinicians’ advice by some patients. Clinicians need to be clear to patients what choices are on offer and be able to provide the rationale for a recommendation.

### Some patients may have treatment preferences for non-clinical reasons

Clinicians may also need to consider the extent to which they would support a choice they believe to be suboptimal (such as TAVI where SAVR would be the standard offer). For example, an issue highlighted in some interviews was the importance of caring responsibilities. In one case, an interviewee declined the trial partly because of their need to look after grandchildren; in another, someone declined a mechanical valve because the warfarin regime would be hard to manage with their other family responsibilities. In other interviews, caring responsibilities arose as an issue in patients who were offered TAVI; it was possible that these patients may not have accepted SAVR because of the longer recovery period.

### Knowledge of the condition and its progress is generally low

Most patients knew little about AS at the time of diagnosis. This improved following diagnosis, particularly where patients used resources from the NHS or heart charities. This meant that patients who were diagnosed with AS some time before developing severe AS were likely to have better knowledge than those who only received an AS diagnosis when it was already severe. In participants in both arms of the RCT, and particularly in the WW group, there was a desire for accurate knowledge about the progression of AS from asymptomatic to symptomatic, including likely time course and what to expect. This difficulty was compounded by the notion of ‘being asymptomatic’, which can be challenging to ascertain, especially as it is dependent on the activity level and comorbidity of the individual.

The findings have a number of practical implications.

1. Early intervention while asymptomatic is unlikely to face many objections in principle from patients. Those who decline are likely to do so for personal and social reasons. Clinicians should note that even if EASY-AS suggests beneficial early intervention, personal and social circumstances can lead patients to decline, underscoring the significant role these factors play in their choices.
2. Where patients are thinking of declining treatment, the offer of choices, such as TAVI rather than SAVR, may help patients.
3. Most patients are aware of the long NHS waiting lists and this may influence their decision-making around early intervention.
4. Patients generally have little knowledge of the condition. A decision-support tool that provides reliable and accurate information and/or links to the many useful resources already available could help.
5. While the study was situated in an RCT, its focus was on factors that would affect decision-making outside that context. As such, its findings may be relevant to other situations where patients are being offered a major intervention for an asymptomatic but serious condition.

## Conclusions

This nested qualitative substudy of an RCT provides important insights into decision-making around having AVR for asymptomatic AS. Patients are generally likely to accept the offer of AVR if recommended by guidelines, although personal circumstances play an important role in decision-making. Where a condition is not well known to the public, such as AS, patients rely on clinicians and other resources for information. Clinicians need to consider individuals’ circumstances, preferences and goals, which may change over time, as well as the importance of accurate information and patient choice, if truly shared decision-making in the management of patients with AS is to be achieved. Liaison with patient groups in developing shared decision-making resources may help with complex decisions.

## Supplementary material

10.1136/bmjopen-2025-106485 online supplemental file 1

## Data Availability

Data are available upon reasonable request.
