# Supplementary material for: Factors influencing the decision to accept or decline aortic valve replacement for asymptomatic aortic stenosis: a nested longitudinal qualitative substudy of the EASY-AS randomised trial
Source: BMJ Open. 2026 Jan 22;16(1):e106485. doi: 10.1136/bmjopen-2025-106485 (PMC12829354; doi:10.1136/bmjopen-2025-106485)
Supplement: online supplemental file 1 [file bmjopen-16-1-s001.docx]

**EASY-AS – EARLY SURGERY GROUP – Interview 1 (within 8 weeks of randomisation) topic guide**

Check you have the right person – i.e., someone who has been randomised to have early surgery – if not, switch to the standard care/ interview topic guide

**Consent – ask to sign written consent form and post, as well as take orally to be transcribed - therefore you need permission to record this section and to start recording from that point**

Check that the interviewee has received and looked at the information sheet. If using Skype or similar for the interview it might be helpful to share a screen and go through the main points. Answer any questions

In going through the information sheet, you will be able to talk about the purpose of the interview and check that the interviewee is happy to have it recorded and transcribed.

You will also emphasise that the interviewee can stop the interview at any point if they don’t want to continue or they simply just want to break.

Check that the interviewee understands how confidentiality is maintained and that any personal details, such as names and specific places, will be removed from the transcript.

You might ask whether the interviewee wants a copy of the report when it’s finished.

**Can you talk me through the process that led to you being diagnosed with Aortic Stenosis?**

Prompts - how did you come to have a diagnosis of aortic stenosis; what was your reaction when you were diagnosed? Were you surprised by the diagnosis (links to next question)?

**What was your understanding of aortic stenosis?**

Prompts - had you heard of it before; did you understand the implications of it? Was anyone particularly helpful or unhelpful when you were getting your diagnosis? In what ways were they helpful? How much time have you invested in finding out about AS since your diagnosis?

**Impact on life?**

Did being diagnosed with aortic stenosis change any aspect of your life: physically socially, relationally, and psychologically? Did you do anything differently to life before diagnosis?

**When making the decision about your treatment for AS, what were the factors that were most important to you?**

Was it living longer, living without symptoms, quality of life, remaining operation-free? Other? What information was most important when making decisions? Did you discuss options with any family members/ friends?

**When considering participating in the EASY-AS trial, how did you feel about the possibility of being referred for surgery when you had no obvious symptoms?**

What were your thoughts on having surgery when asymptomatic? Did you have any preference on which arm of the trial you wanted to be randomised to (and why)? Who else did you discuss with before deciding to take part?

**How did you feel when you were put into the early treatment group?**

Prompts – did you have a preference for early treatment or would you rather have had watchful waiting, did not really mind or are you pleased to be receiving early surgery?

**What are your thoughts about having surgery for AS even though you don’t have any symptoms?**

Prompts – link it to the previous question.

**How much information were you given about what surgery would involve?**

Prompts – were you told whether the surgery would be open-heart or TAVI? Were you given a choice? Were you told about the different types of valve (mechanical or tissue)? If so, were you told about possible advantages or disadvantages of the different types? Were you given a choice? Did you discuss any other choices, like where you might have your surgery or which surgeon or team you would have?

**How has/will being randomised to early treatment affected/affect your life (or your friends and family)?**

Prompts - e.g. travel to surgical centres, cost of travel, disturbance to daily life/family/work, impact on carers, worry about AS/surgery, potential benefits of AS/Surgery?

**What is happening to you now?**

Prompts –are you aware of what the surgery will entail? Have you heard about alternatives forms of surgery? Do you have a preference for less invasive forms of surgery?

**Is there anything you’d like to add?**

Prompts - what advice would you give to anyone in the same position that you were about having surgery when asymptomatic? Would you change anything about the way information about this condition or your treatment options were given to you?

**I’d like to end by asking a few things about you**

- Age
- Marital status
- Live alone or with family, family nearby
- Occupation; current employment status
- Home: rural/urban
- Other illnesses – including mental health issues if participant willing to say
- Length of time since diagnosis of aortic stenosis

**Ending the interview**

- If you haven’t already, ask whether they want a summary or copy of the report
- Thank the interviewee for taking part
