# Supplementary material for: Factors influencing the decision to accept or decline aortic valve replacement for asymptomatic aortic stenosis: a nested longitudinal qualitative substudy of the EASY-AS randomised trial
Source: BMJ Open. 2026 Jan 22;16(1):e106485. doi: 10.1136/bmjopen-2025-106485 (PMC12829354; doi:10.1136/bmjopen-2025-106485)
Supplement: Supplementary file 2 [file bmjopen-2025-106485.reviewer_comments.pdf]

PEER REVIEW HISTORY

BMJ Open publishes all reviews undertaken for accepted manuscripts. Reviewers are asked to complete a checklist review form and are provided with free text boxes to elaborate on their assessment. These free text comments are reproduced below.

ARTICLE DETAILS

Title (Provisional)

Factors Influencing the Decision to Accept or Decline Aortic Valve Replacement for Asymptomatic Aortic Stenosis: A Nested Longitudinal Qualitative Sub-Study of the EASY-AS Randomised Trial

Authors

Allmark, Peter; Taylor, Bethany; Tod, Angela Mary; Ryan, Tony; Dweck, Marc; McCann, Gerry P; Singh, Anvesha

---

VERSION 1 - REVIEW

**Reviewer**	**1**
**Name**	**Nappi, Francesco**
**Affiliation**	**Centre Cardiologique du Nord, Cardiac Surgery**
**Date**	**30-Aug-2025**
**COI**	**None**

I understand that you have dismissed the use of TAVR from the outset. What are the reasons for this?

**Reviewer**	**2**
**Name**	**Zheng, Jianqiao**
**Affiliation**	**Sichuan University West China Hospital, Department of Anesthesiology**
**Date**	**05-Oct-2025**
**COI**	**None**

Dear author:

Thank you for submitting your manuscript “Manuscript ID bmjopen-2025-106485, entitled " Factors Influencing the Decision to Accept or Decline Aortic Valve Replacement for Asymptomatic Aortic Stenosis: A Nested Longitudinal Qualitative Sub-Study of the EASY-AS Randomised Trial." to the BMJ Open. I was honored to comment it as a reviewer.

This study systematically explores the factors influencing the decision of asymptomatic aortic stenosis (AS) patients to accept aortic valve replacement (AVR) through a rigorous longitudinal qualitative design. It fills a gap in clinical research, and its conclusions hold significant guiding value for clinical practice, tool development, and future research. Although there is minor room for improvement in aspects such as sample representativeness and the refinement of conceptual definitions, these limitations do not affect the core value of the study overall.

Publication is recommended. If the figures and tables can be supplemented and improved, and some conceptual definitions refined, the quality of the manuscript will be further enhanced.

1) The study sample was collected from 6 selected centers in the UK. Although the age and gender of the sample are consistent with the population of patients with aortic stenosis (AS), the sample does not cover patients with different socioeconomic backgrounds, educational levels, or ethnicities. Moreover, participants were required to "contact the research team proactively," which may lead to selection bias (e.g., patients who are more proactive and willing to express themselves are more likely to participate).

Suggestions for improvement:

In the "Limitations" section, further explain the geographical and demographic restrictions of the sample, and clearly state that "the results are applicable to patients in similar healthcare systems in the UK, and caution should be exercised when generalizing to other regions."

Briefly mention that future studies could include more diverse samples (such as patients with low educational levels or those living in rural areas) to enhance the generalizability of the results.

2) The study mentions "patients' doubts about being asymptomatic" (e.g., some patients reported symptoms such as fatigue and shortness of breath but were unsure whether these symptoms were caused by AS). However, it does not clearly define the criteria for distinguishing between the clinical definition of "asymptomatic" and patients' subjective perceptions.

Suggestions for improvement:

In the "Methodology" or "Results" section, supplement the explanation that the definition of "asymptomatic" in the study refers to the criteria of the main EASY-AS randomized controlled trial (RCT) (e.g., no clinical symptoms such as dyspnea, chest pain, or syncope). At the same time, record patients' subjective symptom perceptions to clarify the distinction between "clinically asymptomatic" and "subjective discomfort in patients."

3) The manuscript mentions "Figure 1 (Recruitment Flowchart)" and "Figure 2 (Reasons for declining participation in the main EASY-AS trial collected through UK site screening logs)" but does not present the complete figures. Only basic data are marked (e.g., in Figure 2,

"Does not want early AVR/TAVI" is the most common reason, n=101), which affects readers' intuitive understanding of the sample flow and key results.

Suggestions for improvement:

Supplement the complete Figure 1 (which should clearly show the "recruitment path of the four groups of participants," "number of interviews and completion status," and "reasons for loss to follow-up") and Figure 2 (a bar chart is recommended to present the "gender distribution differences in different reasons for refusal"). For example, the original text mentions that the refusal rate of women (33.7%) is higher than that of men (28%), which can be visually compared through the chart.

4) In Figure 2, an orange square has been inadvertently mixed between the blue square (representing sample size) and the gray square (representing males), and this orange square needs to be removed.

**Reviewer**	**3**
**Name**	**bayuo, jonathan**
**Affiliation**	**Hong Kong Polytechnic University, School of Nursing**
**Date**	**02-Dec-2025**
**COI**	**None**

The authors present a longitudinal qualitative study examining the factors Influencing the decision to accept or decline aortic valve replacement for asymptomatic aortic stenosis as a nested study in a randomised trial. Overall, the authors are to be commended for such insightful study which offers interesting findings to support care of patients offered a major intervention whilst asymptomatic. Most importantly, the study had significant input from patients which is relevant. Please see the comments below to help strengthen this paper:

1. The rationale and use of the longitudinal approach remains confusing in the study. For example, there is no mention of how many timepoints were employed for data collection purposes.

2. Further to the above, the longitudinal qualitative stance is employed to examine change that occurs over time which has not been well integrated in this study: how did the patient/ family decisions change across time? While theme 4 (Change over time, page 10) discusses some changes, it is too thin for a longitudinal study. For example, it is not clear the timing of the follow-up interviews, and how often the interviews were carried out. I will recommend a quick look at this paper for some important information about the longitudinal qualitative research approach: Bayuo, J., Nyande, F.K., Awunyo, W. et al. The longitudinal qualitative research design in nursing, health, and social care research: philosophy, methodology, and methods. BMC Med Res Methodol (2025). https://doi.org/10.1186/s12874-025-02736-6

3. Were family members/ next of kins interviewed separately from the patients or interviewed together? The approach employed here will have significant ramifications for the data analysis approach. For example, if they were interviewed together, then it becomes like dyadic interviewing which means analysis is not only about uncovering themes, but also the interactional dynamics. Please ensure to clarify.

4. Why framework analysis and is this approach congruent with the longitudinal stance?

Overall, the study does have some merits, but the methodological choices and their justifications could use more details.

VERSION 1 - AUTHOR RESPONSE

Reviewer-1 :
Dr. Francesco Nappi, Centre Cardiologique du Nord
Comments to the Author:
I understand that you have dismissed the use of TAVR from the outset. What are the reasons for this?
Thank you for your comment. To clarify, we have not dismissed the use of TAVI. All asymptomatic patients with severe aortic stenosis, regardless of whether they were suitable for surgical aortic valve replacement or TAVI, were included in the main EASY-AS trial. This is also stated under the Methods section: 'Participants are randomly assigned in a 1:1 ratio to one of two arms: early intervention ('EI'): AVR or TAVI'. We have now also added the following in the results section: 'Of the 25 interviewees that were also participants of the main randomised trial, 14/25 were assigned EI and 11/25 were assigned to WW. Of the EI group, 3 had TAVIs and the remainder had SAVR based on local clinical team decision'.

Reviewer: 2
Dr. Jianqiao Zheng, Sichuan University West China Hospital
Comments to the Author:
Dear author:
Thank you for submitting your manuscript "Manuscript ID bmjopen-2025-106485, entitled " Factors Influencing the Decision to Accept or Decline Aortic Valve Replacement for Asymptomatic Aortic Stenosis: A Nested Longitudinal Qualitative Sub-Study of the EASY-AS Randomised Trial." to the BMJ Open. I was honored to comment it as a reviewer.

This study systematically explores the factors influencing the decision of asymptomatic aortic stenosis (AS) patients to accept aortic valve replacement (AVR) through a rigorous longitudinal qualitative design. It fills a gap in clinical research, and its conclusions hold significant guiding value for clinical practice, tool development, and future research. Although there is minor room for improvement in aspects such as sample representativeness and the refinement of conceptual definitions, these limitations do not affect the core value of the study overall.
Publication is recommended. If the figures and tables can be supplemented and improved, and some conceptual definitions refined, the quality of the manuscript will be further enhanced.
Thank you for the positive comments on our study and the constructive feedback below.

1) The study sample was collected from 6 selected centers in the UK. Although the age and gender of the sample are consistent with the population of patients with aortic stenosis (AS), the sample does not cover patients with different socioeconomic backgrounds, educational levels, or ethnicities.

Moreover, participants were required to "contact the research team proactively," which may lead to selection bias (e.g., patients who are more proactive and willing to express themselves are more likely to participate).
Suggestions for improvement:
In the "Limitations" section, further explain the geographical and demographic restrictions of the sample, and clearly state that "the results are applicable to patients in similar healthcare systems in the UK, and caution should be exercised when generalizing to other regions."
Briefly mention that future studies could include more diverse samples (such as patients with low educational levels or those living in rural areas) to enhance the generalizability of the results.
Thank you for highlighting the inherent limitation of a study of this scale and design. We have changed the layout in line with the requirements of BMJ Open. There is now a "Strengths and Limitations" section below the Abstract, where we have added two bullet points addressing the points above.

2) The study mentions "patients' doubts about being asymptomatic" (e.g., some patients reported symptoms such as fatigue and shortness of breath but were unsure whether these symptoms were caused by AS). However, it does not clearly define the criteria for distinguishing between the clinical definition of "asymptomatic" and patients' subjective perceptions.
Suggestions for improvement:
In the "Methodology" or "Results" section, supplement the explanation that the definition of "asymptomatic" in the study refers to the criteria of the main EASY-AS randomized controlled trial (RCT) (e.g., no clinical symptoms such as dyspnea, chest pain, or syncope). At the same time, record patients' subjective symptom perceptions to clarify the distinction between "clinically asymptomatic" and "subjective discomfort in patients."
The findings within our study around the uncertainty of being truly 'asymptomatic' is reflective of the inherent difficulty in assessing this in clinical practice, especially in an older comorbid cohort. However, we have clarified in the Methods section that all participants were deemed to be asymptomatic from AS perspective by the recruiting clinicians, i.e., they did not have exertional chest pain, dizziness, syncope or significant dyspnoea thought to be related to AS. In other words, they met the inclusion criteria for the main EASY-AS trial.

3) The manuscript mentions "Figure 1 (Recruitment Flowchart)" and "Figure 2 (Reasons for declining participation in the main EASY-AS trial collected through UK site screening logs)" but does not present the complete figures. Only basic data are marked (e.g., in Figure 2, "Does not want early AVR/TAVI" is the most common reason, n=101), which affects readers' intuitive understanding of the sample flow and key results.
Suggestions for improvement:
Supplement the complete Figure 1 (which should clearly show the "recruitment path of the four groups of participants," "number of interviews and completion status," and "reasons for loss to follow-up") and Figure 2 (a bar chart is recommended to present the "gender distribution differences in different reasons for refusal"). For example, the original text mentions that the refusal rate of women (33.7%) is higher than that of men (28%), which can be visually compared through the chart.
For clarification, Figure-1 refers to the patients recruited for this qualitative sub-study, with number of interviews performed in each group shown in brackets, including numbers with follow-up interviews. The reasons for loss to follow-up have now been added in the main manuscript to avoid the flowchart becoming more confusion. Of the 6 follow-up interviews not conducted, 1 patient withdrew from the main study, 1 from the sub-study before the final interview, and the remainder had surgery too late to allow follow up interview within the required timeframe. Figure-2 is showing summary data from the main randomised trial's screening log for the UK sites, and includes the main reasons given for not wanting to participate in the main RCT (not the qualitative sub-study), and more comprehensive data on this is not available. We have included this information to provide

complementary information for readers for possible reasons people may decline early intervention, as we only interviewed 7 such patients in the qualitative sub-study (though this number is reasonable for such a cohort).

We have changed Figure-2 to a bar chart as suggested, for ease of interpretation.

4) In Figure 2, an orange square has been inadvertently mixed between the blue square (representing sample size) and the gray square (representing males), and this orange square needs to be removed.
A new Figure-2 has now replaced the original one.

Reviewer: 3
Mr. jonathan bayuo, Hong Kong Polytechnic University
Comments to the Author:
The authors present a longitudinal qualitative study examining the factors Influencing the decision to accept or decline aortic valve replacement for asymptomatic aortic stenosis as a nested study in a randomised trial. Overall, the authors are to be commended for such insightful study which offers interesting findings to support care of patients offered a major intervention whilst asymptomatic. Most importantly, the study had significant input from patients which is relevant. Please see the comments below to help strengthen this paper:
Thank you for your positive comments and the useful suggestions below.

1. The rationale and use of the longitudinal approach remains confusing in the study. For example, there is no mention of how many timepoints were employed for data collection purposes.
Apologies that this was not clear earlier. The planned study methodology with the longitudinal timepoints for each group is shown in the flowchart in Figure-1. More details have now been added to the 'Participant selection and recruitment' section of the Methods.

2. Further to the above, the longitudinal qualitative stance is employed to examine change that occurs over time which has not been well integrated in this study: how did the patient/ family decisions change across time? While theme 4 (Change over time, page 10) discusses some changes, it is too thin for a longitudinal study. For example, it is not clear the timing of the follow-up interviews, and how often the interviews were carried out. I will recommend a quick look at this paper for some important information about the longitudinal qualitative research approach: Bayuo, J., Nyande, F.K., Awunyo, W. et al. The longitudinal qualitative research design in nursing, health, and social care research: philosophy, methodology, and methods. BMC Med Res Methodol (2025). https://doi.org/10.1186/s12874-025-02736-6
The frequency and timepoints for the longitudinal interviews for each group has been further clarified (as above), in addition to being displayed in Figure-1. We have also added some clarification under 'Data Analysis' and 'Results' sections. Thank you for the above reference, which we have also added to our manuscript, to clarify the focus of this study. Our main focus was not to examine the change over time, but rather, to use the follow-up interviews with the participants to supplement and triangulate the findings.

3. Were family members/ next of kins interviewed separately from the patients or interviewed together? The approach employed here will have significant ramifications for the data analysis approach. For example, if they were interviewed together, then it becomes like dyadic interviewing which means analysis is not only about uncovering themes, but also the interactional dynamics. Please ensure to clarify.
Thank you for highlighting this. The family members were interviewed separately, and this has been added to the 'Participant selection and recruitment' section.

4. Why framework analysis and is this approach congruent with the longitudinal stance?
Overall, the study does have some merits, but the methodological choices and their justifications could use more details.
Thank you for this. As per our response to comment-2 above, the longitudinal change is not the main focus of this study, and this has been clarified in the manuscript.

VERSION 2 - REVIEW

Reviewer 2

Name Zheng, Jianqiao

Affiliation Sichuan University West China Hospital, Department of Anesthesiology

Date 21-Dec-2025

COI

The revised manuscript has fully addressed the comments from previous reviewers and the journal editor. It has supplemented and refined core definitions, research design details, and figure presentation. Overall, it meets the publication standards and represents a high-quality qualitative research paper.

Reviewer 3

Name bayuo, jonathan

Affiliation Hong Kong Polytechnic University, School of Nursing

Date 15-Dec-2025

COI

Thanks to the authors for clarifying and addressing the concerns raised.
